# Flat-Top Cylinder Indenter for Mechanical Characterization: A Report of Industrial Applications

**DOI:** 10.3390/ma14071742

**Published:** 2021-04-01

**Authors:** Roberto Montanari, Alessandra Varone

**Affiliations:** Department of Industrial Engineering, University of Rome “Tor Vergata”, 00133 Rome, Italy; alessandra.varone@uniroma2.it

**Keywords:** indentation test, FIMEC, material characterization, mechanical properties, industrial application

## Abstract

FIMEC (flat-top cylinder indenter for mechanical characterisation) is an instrumented indentation test employing a cylindrical punch. It has been used to determine the mechanical properties of metallic materials in several applications of industrial interest. This work briefly describes the technique and the theory of indentation with a flat-ended punch. The flat indentation of metals has been investigated through experimental tests, and an equation has been derived to calculate the yield stress from the experimental data in deep indentation. The approach is supported by many data on various metals and alloys. Some selected case studies are presented in the paper: (i) crank manufacturing through pin squeeze casting; (ii) the evaluation of the local mechanical properties in a carter of complex geometry; (iii) the qualification of Al billets for extrusion; (iv) stress–relaxation tests on CuCrZr heat sinks; (v) the characterization of thick W coatings on CuCrZr alloy; (vi) the measure of the local mechanical properties of the molten-zone (MZ) and the heat-affected zone (HAZ) in welded joints. The case studies demonstrate the great versatility of the FIMEC test which provides information not available by employing conventional experimental techniques such as tensile, bending, and hardness tests. On the basis of theoretical knowledge and large amount of experimental data, FIMEC has become a mature technique for application on a large scale in industrial practice.

## 1. Introduction

FIMEC (flat-top cylinder indenter for mechanical characterisation) is an instrumented indentation test that was originally developed for measuring the mechanical properties of irradiated materials, in particular the candidates for applications in future nuclear fusion reactors [1,2]. In this case materials selection is a challenging task because their neutron energy spectrum has a hard component peaked at 14 MeV, not present in the spectrum of fission nuclear reactors. For long time the availability of such neutron source has been recognized as a primary necessity in the community involved in the development of materials for fusion reactors; now, a D-Li neutron source (IFMIF) is under development. Its mission is to produce high energy neutrons at sufficient intensity and irradiation volume to test samples of candidate materials. The relatively small volume of the IFMIF irradiation chamber (about 500 cm^3^ for the higher fluence zone of about 20 dpa/year), the large number of specimens required for the mechanical characterization of irradiated materials, the strong gradient of the irradiation flux, as well as the high irradiation cost impose the use of miniaturized probes. The mechanical properties (dynamic fracture toughness, tensile, creep, stress-relaxation, fatigue etc.) should be determined by using miniaturized probes. Data obtained from such tests differ in general from those of standard mechanical tests. To assess bulk properties, it is necessary to use empirical expressions that represent a serious drawback.

A cylindrical punch of sintered tungsten carbide (diameter = 1 mm and axial length = 1.5 mm) is commonly employed in FIMEC test; punches of a smaller size (down to 0.5 mm) are also used, depending on the material characteristics and the zone examined. The method allows one to perform several tests on a small volume of material; thus, it is of great interest for the characterization of irradiated materials [3,4].

The test provides applied load vs. penetration depth curves and can be performed at different temperatures from −196 °C up to +600 °C. The properties which can be determined from FIMEC test are yield stress [1], elastic modulus [5,6], ductile to brittle transition temperature (DBTT) [2,4], surface creep [1] and stress relaxation [6,7]. The experimental apparatus and data processing are quite simple; the test is non-destructive and the local properties of mechanical parts of complex geometry can be easily determined, including those of the molten zone (MZ) and the heat-affected zone (HAZ) in welded joints [8,9,10]. In comparison with other indentation tests operating on a micro- and nano-scale, a clear advantage is that the FIMEC results are scarcely affected by surface conditions (roughness, chemical segregation etc.). Moreover, owing to the punch size thousands of grains are involved in each single indentation; thus, the results are statistically representative of the whole material.

The characteristics of indentation tests with cylindrical indenter have been exhaustively described in the review paper of Yang and Li [11]. Compared with indenters of other shape, the great advantage of the flat indenter is that the material quickly reaches a fully plastic state, and the size of the plastic zone under the indenter does not change during further indentation [1,6,12]. Owing to the aforesaid characteristics FIMEC test has proved to be very useful for several industrial applications. For instance, it has been used to study the local mechanical properties of aluminium die cast large components [13], the stiffness reduction of CFRP (Carbon Fiber Reinforced Polymer) composites after different ageing in water [14] and the water uptake of coated and uncoated CFRPs [15].

Xu et al. [16] used flat cylindrical indentation to determine the creep behavior of Ni-based single crystal superalloys. An indentation technique with a nearly flat tip indenter was used by Midawi et al. [17] to measure the anisotropy of yield strength in API-X80 line pipe welds, while Kim et al. [18] derived fracture toughness by assuming that the load–depth curve of the indentation test is the same as the load–displacement curve of the Cracked Round Bar (CRB) test. Flat-ended punch nanoindentation has been used to determine the interface strength in brittle matrix composites through single fiber push out tests [19] and to study the mechanical behavior of carbon–carbon composites [20] and thin films [21]. On one hand there is an increasing use of flat-ended indentation to study the characteristics of a large variety of materials, on the other hand the theory has been developed to include the effects due to indenter size.

This work aims to demonstrate that the technique is mature for an industrial use on a large scale in different fields. After a brief review of the theoretical models developed to account for the cylindrical indentation, the work reports some selected case studies showing the versatility and efficiency of FIMEC test applied to problems of industrial interest. Specifically, the following applications have been described: (i) crank manufacturing through pin squeeze casting; (ii) evaluation of the local mechanical properties in a carter of complex geometry; (iii) qualification of aluminium billets for extrusion; (iv) stress–relaxation tests on CuCrZr heat sinks; (v) characterization of thick tungsten coatings on CuCrZr alloy; (vi) measure of the local mechanical properties of MZ and HAZ in welded joints.

## 2. Cylindrical Punch Indentation: Theory and Experiments

**Figure 1:**
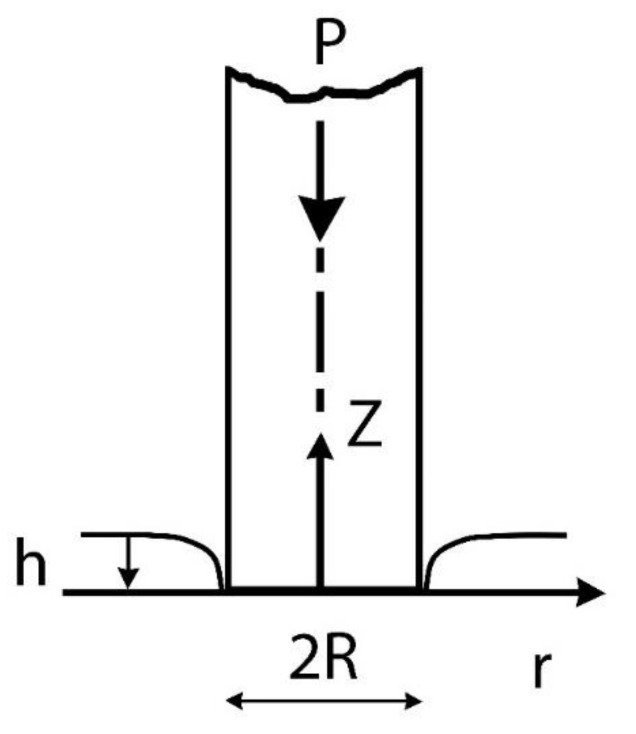
Schematic view of indentation by a flat-ended punch.

Following the pioneering work of Hertz [22], Cerruti [23] and Boussinesq [24] found the solution to the problem of an elastic half-space subjected to a pressure acting on a closed surface by using the potential theory method. Love [25] determined the solution for conical and cylindrical indenters, while Sneddon [26] derived the load-displacement relations for an arbitrary shaped axisymmetric punch. In his work, Sneddon considered the frictionless indentation of an elastic half-space by a flat-ended cylindrical punch, and the contact area is assumed to be equal to the indenter tip area of radius *R.* The boundary conditions in the local system *r*-*z* (Figure 1) can be expressed as

*σ_z_* (r,0) = 0       *r* > *R* *τ_rz_* (r,0) = 0     0 ≤ *r* ≤ *R* *u*_z_ (r,0) = *h*     0 ≤ *r* ≤ *R* (1)

No normal stress *σ_z_* acts on the free surface outside the contact region (first condition); there is no friction between indenter surface and half space (second condition); the displacement *u_z_* in *z* direction is consistent with the flat end of the punch (third condition).

The punch has a sharp edge thus σ*_z_* → ∞ when *r* = *R* and localized plastic deformation occurs on the circular edge [27]. Owing to the constant contact area, the relationship between the mean contact pressure *P_m_* and the penetration depth *h* is linear and given by:

(2) $$P_{m\;}=\frac{2Eh}{\pi R\left(1-{\upsilon \;}^{2}\right)},$$

*E* being the Young’s modulus, and *υ* the Poisson’s ratio.

According to the Sneddon’s solution [26,28], the stress field in the elastic half-space can be expressed in the (*r*, *θ*, *z*) cylindrical coordinate system by the following equations:

(3) $$\sigma_{r}=-\frac{1}{2}P_{m}\left\{\left[-z\frac{d^{2}}{dz^{2}}-\frac{d}{dz}\right]\arctan\left[\frac{1+K\sin\Phi }{z+K\cos\Phi }\right]-\frac{1}{r^{2}}\left[\left(1-2\nu \right)+z\frac{d}{dz}\right]\left(1-K\sin\Phi \right)\right\},\;\sigma_{\vartheta }=P_{m}\left(1+\nu \right)\frac{d}{dz}\arctan\left[\frac{1+K\sin\Phi }{z+K\cos\Phi }\right]-\sigma_{r}-\sigma_{z},\;\sigma_{z}=-\frac{1}{2}P_{m}\left[z\frac{d^{2}}{dz^{2}}-\frac{d}{dz}\right]\arctan\left[\frac{1+K\sin\Phi }{z+K\cos\Phi }\right],\;\tau_{rz}=-\frac{1}{2}P_{m}\frac{z}{r}\frac{d^{2}}{dz^{2}}\left(1-K\sin\Phi \right),$$

where *R* is assumed to be equal to 1, *K*^4^ = (*r*^2^ + *z*^2^ − 1) + 4*z*^2^ and

$$\tan\left(2\Phi \right)=\frac{2z}{r^{2}+z^{2}-1}$$

If plasticity is included in the indentation model, the constitutive equations are not linear and involve some parameters of the material (yield stress and work-hardening coefficient) for studying its plastic behavior. Different approaches have been used to describe the stress–strain field induced during indentation by punches of different shapes; the spherical cavity [29] and the slip-line field [30] models are the most relevant ones.

Under the condition that the material follows the Tresca plasticity criterion, Shield [31] showed that the axisymmetric plastic flow of a rigid-plastic material can be described by a slip line field and determined the stress field for the indentation of a semi-infinite solid by a flat-ended cylindrical punch. The mean contact pressure *P_m_* was estimated to be 5.69 *ξ* (*ξ* is the shear strength of the material); the maximum contact pressure *P_max_* (close to punch edge) is 7.2 *ξ;* and the radial extension of the plastic area is 1.58 *R*. A further work of Eason and Shield [32] demonstrated that plastic deformation starts at punch edge, and then it progressively extends with penetration depth and reaches the indenter axis when *P_m_* is about six times the shear strength, namely, three times the tensile yield stress σ_Y_, as a consequence of the Tresca plasticity criterion.

The FIMEC apparatus was designed and realized at the beginning of 90s, and the first results were published in 1994 [1]. At that time cylindrical indentation was not as popular as it is today. In addition to the aforesaid theoretical studies, the work of Yu et al. [33] was of particular importance for developing the technique. These investigators determined some empirical formulae differentiating the load–penetration curve from a conventional compression test. Then the effects of the round corners of a flat punch in an elastic–plastic field were investigated by Ciavarella et al. [34]. Scibetta et al. [35] analyzed data from experiments on several materials through a simplified formula that gives the constraint factor as a function of the hardening exponent and the limit value determined by Shield for rigid–plastic materials.

The flat indentation of metals was investigated by us through experimental tests and FEM providing an interpolation formula for the load–penetration relationship [6]. Such work was further developed, and a new equation has been derived to calculate the yield stress from the experimental data in deep indentation [36]. Across 25 of years activity, a lot of experimental data have been collected on various metals and alloys which support this approach. Recently, some of these data were used by Brutti [37] to validate a new theoretical model that allows one to solve the direct and the inverse problems of flat indentation. Moreover, FIMEC test has been successfully used in different industrial applications; some examples of case studies are reported in this paper.

**Figure 2:**
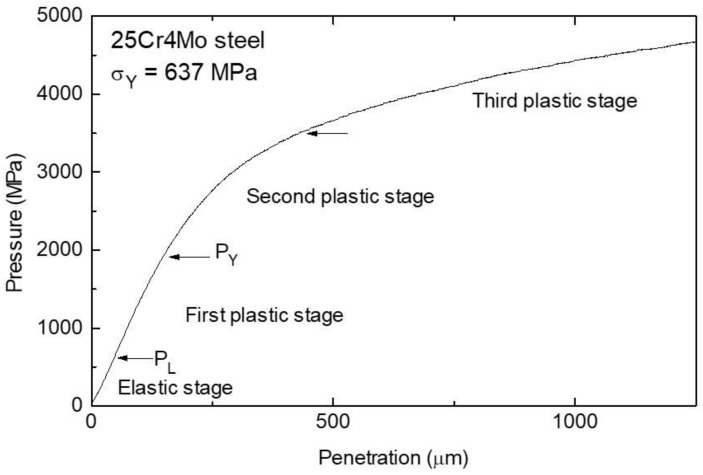
Flat-top cylinder indenter for mechanical characterisation (FIMEC) indentation curve of 25Cr4Mo steel.

**Figure 3:**
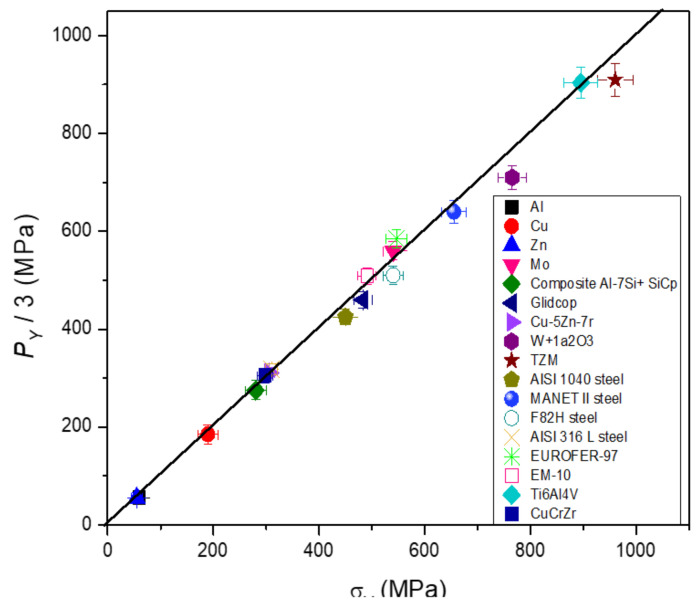
P_Y_ /3 from FIMEC test are plotted vs. σ_Y_ data from tensile tests of different metals and alloys. All the points are close to the bisector line showing that σ_Y_ is approximatively equal to P_Y_/3.

A typical curve obtained from FIMEC test is displayed in Figure 2. After an initial elastic stage up to a pressure P_L_, three plastic stages are observed: (i) the first one is nearly linear and ends at P_Y_; the second one starts when the plastic deformation reaches the indenter axis (P > P_Y_) and exhibits a curve slope decrease; the third stage has a trend with an almost constant slope. More details are reported in ref. [6]. On these grounds, it is possible to directly compare data from FIMEC indentation and tensile tests: the yield stress σ_Y_ is approximately equal to P_Y_/3. This is confirmed by a lot of experimental evidence, e.g., Figure 3 displays P_Y_/3 vs. σ_Y_ data of different metals and alloys, and all the points lie close to the bisector line.

The relationship σ_Y_ = P_Y_/3, which is analogous to the findings of Eason and Shield [32] theoretical work, has been assessed to be valid by testing different pure metals and alloys also at high and low temperature [6]. Of course, the critical point is the accurate determination of the point P_Y_ in the load–penetration FIMEC curve from which the yield stress σ_Y_ is then calculated. In the second and third stages, P can be described by a relationship similar to that often used for modelling stress-strain curves:

P = *K* (*h*_0_ + *h*)*^n^* (4)

where *K* and *h*_0_ are constants, *n* the strain-hardening exponent. Equation (4) allows one to fit the FIMEC curves in the secnd and third stages and determine the point P_Y_. The method has been described in detail in [36], and the results show that the relative difference between P_Y_/3 and σ_Y_ obtained in tensile tests does not exceed ±7%, namely, the σ_Y_ data scattering resulting from tensile tests on the same material [38].

An experimental parameter in FIMEC tests that must be properly chosen is the indentation rate because it affects the trend of pressure–penetration curves [1]. The relationship σ_Y_ = P_Y_/3 is applicable only in the case that the indentation tests are carried out with a penetration rate of 0.1 mm/min or lower [6].

FIMEC tests can be made by employing punches of diameter in the range 0.5–1 mm depending on the characteristics of the mechanical parts to examine; the pressure–penetration curves obtained by testing the same material show an almost perfect overlapping [5].

Recently, the large use of small components in many industrial fields stimulated the research on test methods employing indenters of very small size; however, such effort encountered a serious problem first evidenced by Nix and Gao [39]: the measured hardness of a material has been found to increase as the size of the indenter decreases. The phenomenon has been termed indentation size effect (ISE). Campbell and Gill [40] proposed a model for flat indentation taking into account ISE that reproduces the force–displacement loading and unloading response of flat punches of different widths. Their model shows good agreement with experimental data (e.g., [41]) and has the following main characteristics: (i) the contact pressure depends on two length scales (punch width and indentation depth); (ii) shape and connectivity of the plastic zones change with indentation depth and punch width; (iii) the proportion of the deformation accommodated by elasticity and plasticity changes.

## 3. Case Studies

### 3.1. Crank Manufacturing through Pin Squeeze Casting

Pressure die casting is a common manufacturing process used in many industrial sectors, in particular those with strong automation such as the automotive one. Today, manufacturers tend to produce components by using increasingly higher pressures with the scope to increase the size of components and reduce the residual porosity. Pressure increase involves higher cooling rates of castings that lead to thermal gradients and shape distortion of the manufactured components. Therefore, the industrial process where castings are submitted to the highest pressures (squeeze casting) is still a niche process. An intermediate solution between squeeze casting and die casting is pin squeeze casting where the highest pressure is applied only in some areas of the components through small pins before the complete solidification of casting when the material is semi-solid.

**Figure 4:**
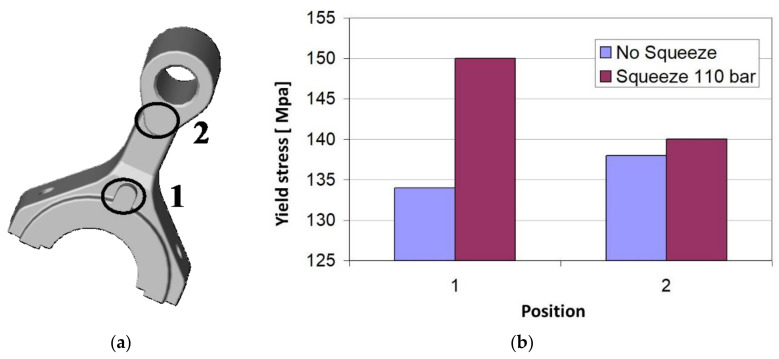
Yield stress determined through FIMEC test in the points 1 and 2 of the crank (**a**) produced with and without local pressure of the pin in the point 1 (**b**).

In the case considered here, pin squeeze casting has been employed for improving the mechanical properties of cranks of small size made of the aluminum EN-AB46000 alloy and used in compressors of domestic refrigerators. In order to establish the optimal process conditions, the knowledge of local mechanical properties of cranks was of the utmost importance, in particular in the zones subjected to the higher stresses in service life (point 1 in Figure 4a).

FIMEC tests were performed in these points of cranks produced with and without local pressure (110 bar) of the pin. The additional pin pressure has been applied on the opposite side of that subjected to FIMEC indentation test; the measured values of yield stress are displayed in Figure 4b.

The local pin pressure has two remarkable effects. The first effect is to increase the yield stress in the whole crank (also in point 2 far from the zone where pin pressure is applied) to values which cannot be reached in the ordinary die casting process. The second one consists of the change of the local mechanical properties: in the crank produced without the pin pressure the yield stress is higher in point 2 than in point 1, whereas the contrary occurs if additional pin pressure is applied in point 1 during the process. This is very important because pin squeeze casting guarantees the best mechanical properties in the zone of the crank that is subjected to the highest stress in exercise.

### 3.2. Evaluation of the Local Mechanical Properties in a Carter of Complex Geometry

**Figure 5:**
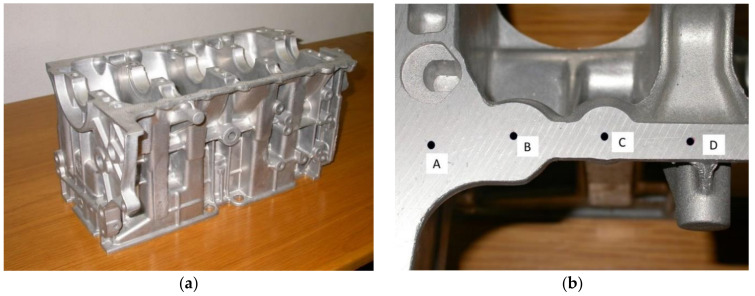
Carter in EN-AB46100 alloy (**a**). Several FIMEC tests have been performed in different positions (A, B, C, D) of this mechanical component to evaluate the local mechanical properties (**b**).

Components of a complex shape are commonly produced through foundry processes and non-homogeneous cooling rates often give rise to different local mechanical characteristics in different zones of the same piece. The case presented here is that of a carter (Figure 5a) made in aluminum EN-AB46100 alloy. About one hundred FIMEC tests have been performed in different areas of the carter (e.g., see Figure 5b) to determine the local yield stress, and on this basis the die sections have been suitably modified to obtain more homogeneous mechanical properties.

To perform such measurements the experimental apparatus has been modified due to the great size of the tested component. The standard FIMEC apparatus operates through a linear actuator (an electro-mechanical drive equipped with a step motor) [6]. The motor rotation is transmitted to a ball screw that converts rotation into translation, guided by means of a pre-loaded ballspline, and the indenter is mounted at the end of a rod while the load cell is allocated under the sample holder. For carrying out these measures, the load cell has been mounted between the linear actuator and the tested piece.

### 3.3. Qualification of Aluminium Billets for Extrusion

In manufacturing mechanical parts by extrusion, the control of process parameters (applied stress and deformation rate) is of the utmost importance to obtain products with standard and reproducible characteristics. A problem often encountered by forgers is connected to the quality and properties of the billets which are commonly provided by different suppliers. Billets of the same material with controlled chemical composition may give rise to quite different extruded products if the microstructure is different, in particular the size and orientation of crystalline grains along the billet thickness plays a critical role. Therefore, the mechanical properties of the billets should be assessed before extrusion to avoid manufacturing rejects and higher costs.

The FIMEC test has revealed to be a technique that guarantees a fast response and sound results in testing the billets to be employed in the extrusion process. It allows one to obtain a map of yield stress along the thickness of the billet and is helpful in the choice of suitable process parameters.

**Figure 6:**
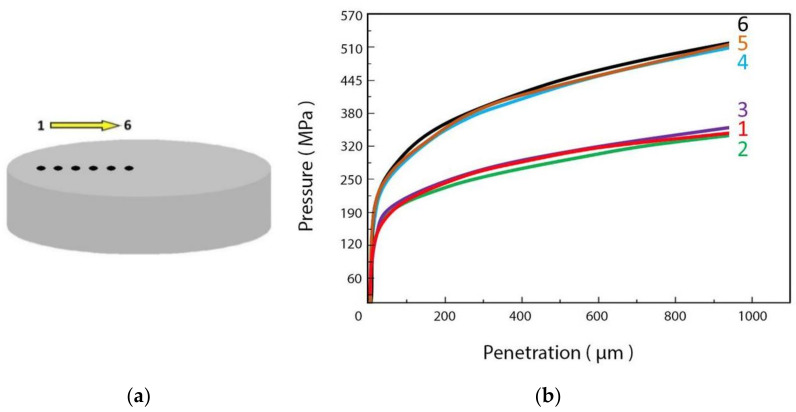
Schematic view of a section of an aluminium billet where FIMEC tests were performed in different positions along the radial direction (**a**). The pressure–penetration depth curves are displayed in (**b**).

For example, Figure 6a displays the section of a billet and the positions where indentation tests have been performed; the pressure-indentation depth curves in (b) clearly show that the mechanical properties change along the radial direction. Once the optimal characteristics for extrusion under standard work parameters have been identified, FIMEC tests allow one to check whether a given billet stock is suitable to be processed.

### 3.4. Stress-Relaxation Tests on CuCrZr Heat Sinks

**Figure 7:**
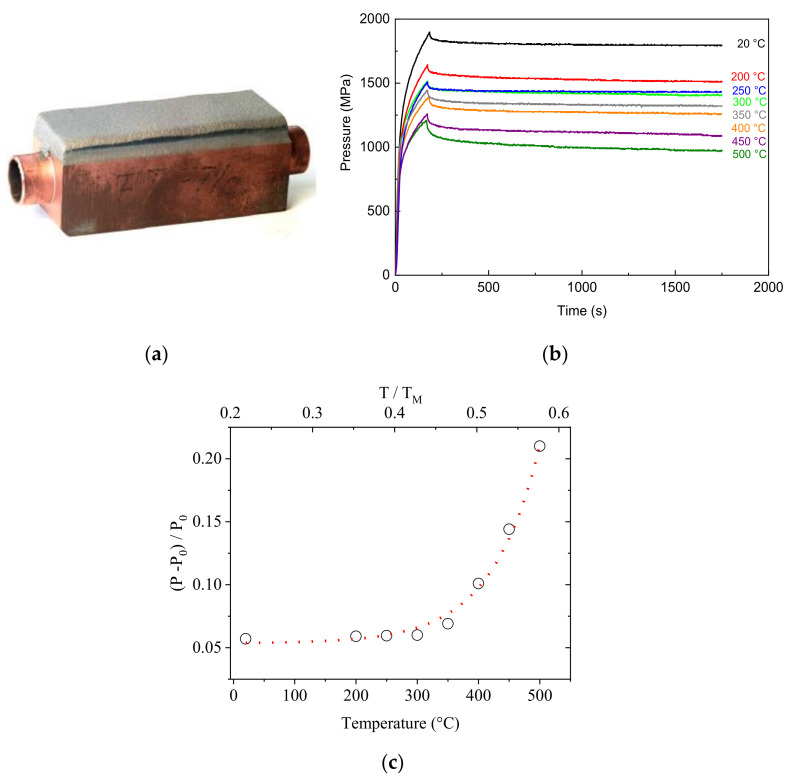
FIMEC stress relaxation tests on CuCrZr alloy: heat sink (**a**); pressure vs. time curves at increasing temperatures (**b**); relative variation of the stress *P* with respect the initial value *P*_0_, −*P* − *P*_0_)/*P*_0_, after 1750 s (**c**).

The CuCrZr copper alloy is a promising material for high heat flux applications in future fusion reactors such as the International Thermonuclear Experimental Reactor (ITER), in particular for manufacturing heat sinks which are commonly protected from plasma by W armors. For example, Figure 7a displays a component with a thick W coating on one face. During a long-term operation, thermal creep and stress relaxation cause permanent damage to the material at elevated temperature and stress levels below the yield point; thus, these phenomena have been extensively investigated (e.g., see ref. [42,43,44,45]).

The FIMEC indentation test has been used by us to investigate stress relaxation at different temperatures up to 500 °C, namely, above those foreseen in exercise (≈300 °C). The scope was to identify the critical temperature above which stress relaxation represents a serious problem for the components.

The examined alloy was supplied by Outokumpu (Jyväskylä, Finland) and had the following composition: Cr 0.73, Zr 0.14, Cu to balance (wt%). The stress–relaxation tests have been carried out by stopping the penetration and recording the evolution of stress vs. time, while the penetration depth was kept constant. The curves obtained in tests at increasing temperature are reported in Figure 6b. After an initial drop, the stress exhibits an exponential trend which can be described by the following relationship:

(5) $$P=P_{0}e^{-t/\tau }$$

Relaxation consists of the progressive transformation of elastic deformation into plastic deformation thus, since the sum of elastic and plastic deformation is constant, the measured stress *P* decreases. Data in Figure 7b clearly show a strong test temperature dependence. The relative variation of the stress *P* with respect the initial value *P*_0_, (*P* − *P*_0_)/*P*_0_, after 1750 s is reported in Figure 7c and shows how there is a significant increase when temperature exceeds 0.45 T_M_ (T_M_ is the melting point) corresponding to about 330 °C, when the typical mechanisms of high temperature deformation (climb and dislocation cross-slip) become active. The result evidences how the critical temperature for stress relaxation is just a little above the maximum one foreseen in service for CuCrZr components; therefore, great attention should be paid to the temperature fluctuations involved in a system operating in pulsed mode.

### 3.5. Characterization of Thick W Coatings on CuCrZr Alloy

As mentioned in the previous section, W is the first-choice material to protect structural components, divertor and cooling systems in future nuclear fusion reactors because of its excellent thermo-mechanical proprieties, high melting point, high thermal conductivity, low physical sputtering, tritium retention, and activation under neutron irradiation [46,47,48].

**Figure 8:**
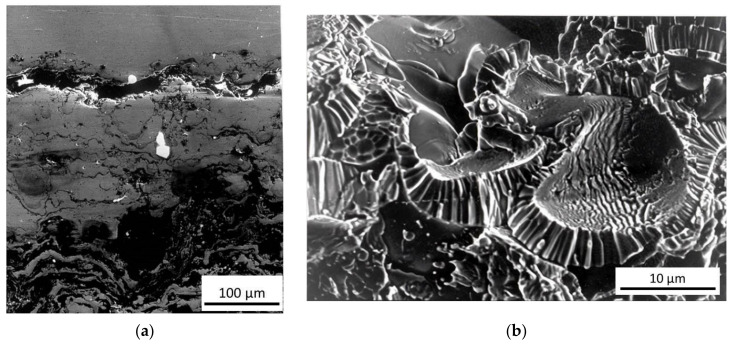
Morphology of the W-CuCrZr interface (**a**); fracture surface of plasma sprayed W showing the structure with columnar grains (**b**) [51].

An option is the use of bulk W tiles; however, the poor W machinability is a serious drawback for realizing parts of complex geometry, e.g., those for protecting tubes and pipe fittings of the cooling system. An alternative solution is the deposition of thick coatings on such components commonly made of the CuCrZr alloy. Joining is a hard task for the high thermal expansion mismatch between and CuCrZr (α_Cu_ ≈ 4α_W_), leading to high residual stresses with the possible formation and propagation of cracks and final detachment of the coating. Therefore, a lot of coating technologies have been examined to find the suitable ones, and the attention has been focused on plasma spray (PS) in vacuum or controlled atmosphere [49,50] for its simplicity, low costs, and the possibility to cover large surfaces. W coatings (5 mm-thick) on CuCrZr substrates have been realized by means of PS in controlled atmosphere by Riccardi et al. [51,52], and they were able to withstand heat fluxes up to 5 MW/m^2^. An interlayer with thickness of about 800 µm was deposited between substrate and coating with intermediate thermal expansion coefficient; it consisted of a layer of pure Ni and a successive stratification of layers of grading mixtures of Al–12%, Si, and Ni–20% Al. Figure 8a shows the complex morphology of the interface and the presence of some porosity and unmolten particles which may represent preferred sites for crack nucleation. As displayed in the fractographic image of Figure 8b, the grains of the W coating exhibit a columnar structure which involves strong anisotropic mechanical properties. These characteristics have been investigated through FIMEC indentation tests.

**Figure 9:**
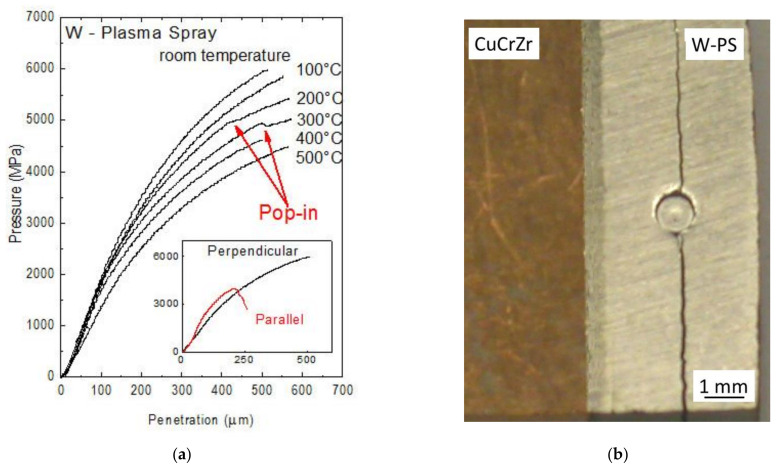
FIMEC curves obtained by indenting the W coating along the direction perpendicular to the surface at increasing temperatures up to 500 °C (**a**). In the frame, the curves obtained in room temperature tests by indenting along perpendicular and parallel directions are compared. A long crack induced by parallel indentation is shown in (**b**) [52].

Figure 9a displays the FIMEC curves obtained by indenting the W coating along the direction perpendicular to the surface at increasing temperature up to 500 °C [52]. Even if some curves (e.g., at 200 and 300 °C) exhibit pop-ins due to the formation of small cracks during punch penetration, in general the material has a good resistance. The values of yield stress are reported in Table 1.

On the contrary, when indentation is carried out along the direction parallel to the coating surface, long cracks form at the beginning of the penetration test inducing a pressure drop. The curves obtained in the room temperature tests by indenting the W coating along the two directions (perpendicular and parallel) are compared in the frame of Figure 9a, while a long crack induced by parallel indentation is shown in Figure 9b. The results indicate a strong anisotropic mechanical behavior of the coating strictly connected to the columnar growth of W grains during deposition.

To overcome such drawback, a different approach has been investigated in the last years, i.e., the realization of the interlayer as a functionally graded material obtained by depositing successive layers with varying fractions of Cu and W, i.e., the W content progressively decreases with the distance *x* from the pure W coating [53]. The volume fraction *V_W_* of W in the graded layers is expressed by

(6) $$V_{W}=1-{\left(\frac{x}{t}\right)}^{p}$$

*t* being the thickness, and *p* the gradient index. An actively water-cooled mock-up designed by using such approach showed compressive and homogeneously distributed stresses in the interlayers under operational conditions.

### 3.6. Measure of the Local Mechanical Properties of the MZ and the HAZ in Welded Joints

The quality of welded joints is commonly investigated in the industrial practice through tensile, bending, and micro-hardness tests. Although these techniques are quite useful, they have some limits. Tensile and bending tests carried out on probes with a welded joint provide information on the average behavior of the material but not on the specific mechanical characteristics of the MZ and HAZ, which are of great relevance for optimizing the post-welding heat treatments (PWHT). Moreover, bending tests do not always help in revealing an inadequate structure in the whole welding bead, since the maximum stress is mainly concentrated in the MZ. Micro-hardness profiles across the joints give the local properties of the MZ and HAZ, but data are often quite scattered because they are strongly affected by the specific tested area of the order of tens of microns. The FIMEC test allows one to measure the yield stress in the MZ and HAZ of joints realized through common welding processes such as MIG and TIG, while it is not suitable in the case of electron beam and laser welds because the seams are too thin, smaller than the indenter size. Moreover, since FIMEC imprint involves a large number of grains, the results are representative of the average behavior of the investigated MZ and HAZ. Therefore, it has been often used to investigate the evolution of local properties in the MZ and HAZ of welds of steels [9,10,54] and light alloys [8] before and after PWHTs to find the best treatment conditions.

The first example presented here regards Gas Tungsten Arc Welding (GTAW) of the martensitic stainless steel Eurofer-97 whose composition is reported in Table 2.

**Figure 10:**
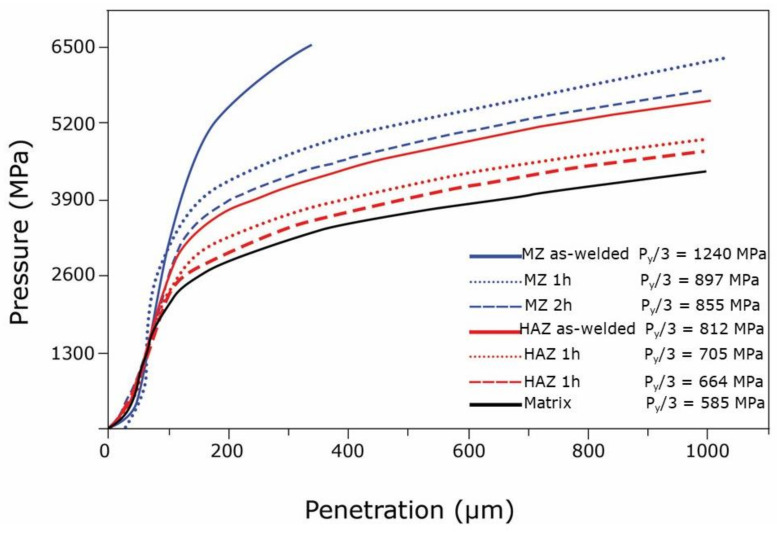
Gas tungsten arc welding (GTAW) joints of Eurofer-97 steel. FIMEC curves obtained from base metal, the HAZ, and the MZ in as-welded condition and after post-welding heat treatments (PWHTs) of 1 and 2 h at 750 °C.

Eurofer-97 steel is insensitive to cold- and hot-cracking; however, as the material is a self-hardening steel, the weld process promotes the formation of martensite in the MZ even with air-cooling, with the result of an unacceptable hard and brittle structure. Therefore, joints need a suitable PWHT that may be optimized to find the best compromise between the recovery of toughness in the MZ and grain coarsening, precipitation, and segregation, which weaken the HAZ. The scope of our investigation was to identify the temperature-time combination of PWHT providing mechanical properties of joints as close as possible to those of the base material. A set of samples was prepared by treating the welds at 700, 730, and 750 °C for 1, 1.5, and 2 h, and FIMEC tests were performed in base metal, the HAZ, and the MZ of each sample. For example, Figure 10 shows the curves obtained in tests carried out on as-welded samples and treated for 1 and 2 h at 750 °C and the corresponding values of yield stress P_Y_/3.

In the as-welded condition, the MZ exhibits a yield stress of 1240 MPa, typical of a fully martensitic structure, while P_Y_/3 measured in HAZ is 812 MPa, an intermediate value between those of the MZ and matrix (585 MPa). The PWHTs reduce P_Y_/3 in both MZ and HAZ in different degree depending on soaking time. The results of FIMEC tests on all the samples indicated that the original properties are never fully recovered in the MZ and HAZ; the treatment of 2 h at 750 °C guarantees the best results.

This PWHT was adopted for the cooling plates of Eurofer-97 steel welded in assembling operations of the Test Blanket Module (TBM) of the International Thermonuclear Experimental Reactor (ITER) [55].

In the second example the FIMEC test has been used to assess the quality of GTAW welded joints of the duplex stainless steel UNS S31803 with biphasic structure. The compositions of steel and filling material supplied by Outokumpu (Jyväskylä, Finland) are reported in Table 3.

**Figure 11:**
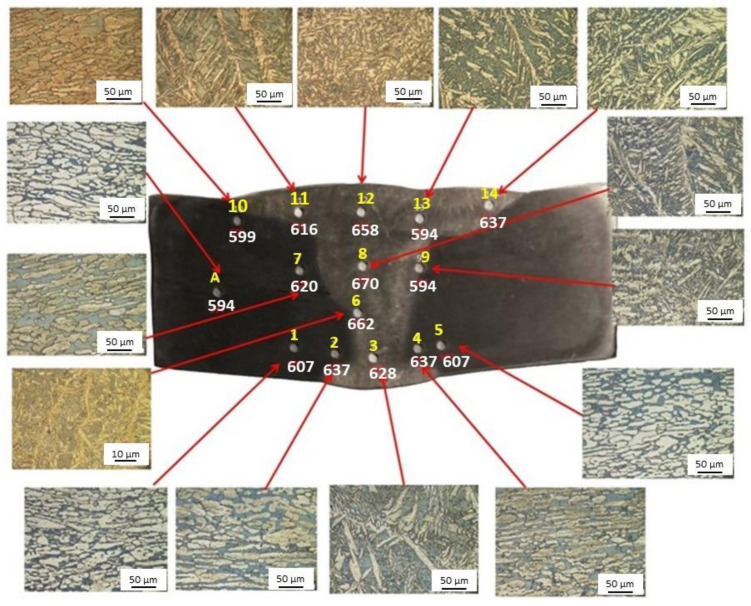
Local microstructure of the joint in areas close to the positions where 15 FIMEC indentation tests were performed (yellow). For each position, the P_Y_/3 values determined from FIMEC tests are reported (white).

After welding, the joints have been heat treated at 1050 °C for 600 s (ASTM A928/A928M [56]). The samples have been then subjected to common metallographic preparation consisting of mechanical polishing and etching by means of the solution of 80 mL H_2_O_2_, 30 mL HCl, and 1 g K_2_S_2_O_5_. A total of 15 FIMEC tests have been made in different positions of each joint as shown in Figure 11, which displays also the corresponding local microstructure.

Table 4 reports the P_Y_/3 values determined by FIMEC tests and the average micro-hardness HV measured in the same positions.

The base metal (point A) exhibits grains of austenite (brighter) and ferrite (darker), whereas in the MZ also grains of austenite with Widmanstätten morphology are observed. The yield stress and micro-hardness variations depend on the microstructural changes observed in the investigated positions. In fact, different heat fluxes in MF and HAZ determine a change of the relative fraction of ferrite and austenite, which affects the local mechanical properties. In general micro-hardness and FIMEC values show a good correspondence because the HV/P_Y_/3 ratio lies in a narrow range (0.43–0.48); however, micro-hardness data are more scattered because each imprint is made either in an austenite grain or in a ferrite grain, whereas many grains of both phases are involved in a single FIMEC test.

An important microstructural aspect that can lead to the relevant variations of mechanical properties in welded duplex stainless steels (DSS) is the precipitation of second phases (SP). This represents a critical issue for industrial processes; therefore, a specific work has been carried out to assess whether data from FIMEC tests can be somehow correlated to SPs precipitation and provide indirect information about such microstructural features. The material used in the experiments was the 2205 steel, one of the most used DSS for applications in the pulp and paper, chemical processing, refining, petrochemical, food and beverage, and transportation industries. The examined material was supplied by Sandmeyer Steel Co (Philadelphia, PA, USA), and its nominal composition is given in Table 5.

The precipitation of SPs was induced by heat treatments at 750, 850, and 900 °C for increasing soaking time (1, 2, 5, 8, and 10 h); the samples were examined through X-ray diffraction and optical and electron microscopy (SEM and TEM) to identify type, fraction, and morphology of SPs, and finally they were tested by FIMEC.

**Figure 12:**
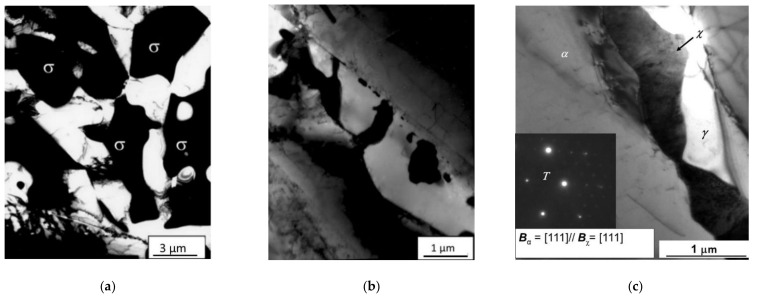
Morphological features of second phases (SPs) in 2205 steel. The σ phase morphology is “oil spot” (**a**) or “coral-like” (**b**) depending on precipitation temperature. The micrograph in (**c**) shows the χ phase and is taken from ref. [54]. From: “Flat-top cylinder indenter examination of duplex stainless steel 2205 after different heat treatments” by G. Angella, A. Fava, R. Montanari, M. Richetta, A. Varone, 2017, *Metals*.

The as-supplied steel has a biphasic structure with approximately equal amounts of austenite γ and ferrite α that guarantees an excellent combination of mechanical properties and corrosion resistance [57]. Following heat treatments, the precipitation of SPs occurs with detrimental effects on mechanical properties; in particular, they cause a relevant toughness decrease [58,59,60]. Among SPs, the most dangerous is the brittle σ phase which forms in the temperature range 600–1000 °C with the fastest kinetics at about 850 °C. Because of its high Cr content, the σ phase depletes the surrounding matrix of this element with a consequent decrease in corrosion resistance. In addition, the linking of σ particles favors the formation of long cracks. Its morphology depends on the precipitation temperature: following heat treatments in the range 850–900 °C, the σ phase becomes more compact and the particles join together giving rise to the “oil spot” morphology (Figure 12a), whereas at lower temperatures (750 °C) it grows in the “coral-like” morphology (Figure 12b). Another intermetallic compound coexisting with σ phase is the χ phase (Figure 12c) that forms at 750 °C, even if in smaller amounts than σ phase [53,59]. The χ particle shown in Figure 12c nucleated at a α/α boundary and the inset shows the crystallographic relationship between the χ and α phases. Nitrides and M_23_C_6_ carbides were also observed in all of the examined samples, but their volume fractions are very small with respect to the other SPs. In fact, microstructural examinations showed that σ phase represents the largest part of SPs present in the steel after all the heat treatments.

**Figure 13:**
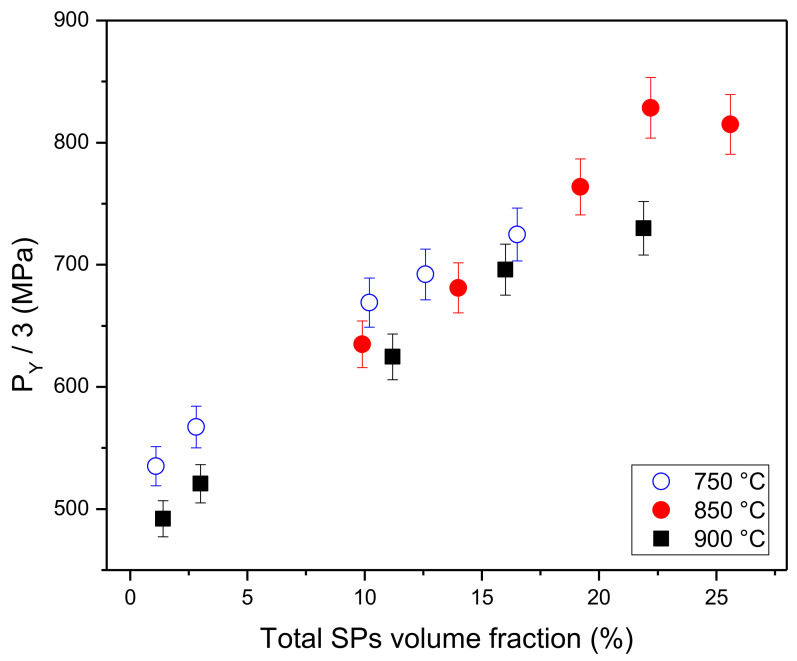
P_Y_/3 values determined from the FIMEC tests vs. total volume fraction of SPs formed after heat treatments at 750, 850, and 900 °C. Redrawn from ref. [54].

Figure 13 displays the P_Y_/3 values determined from FIMEC tests vs. the total volume fraction of SPs developed after heat treatments at 750, 850, and 900 °C.

By comparing these data with the yield stress of as-supplied material (P_Y_/3 = 470 MPa), it is clear that FIMEC allows one to reveal very small amounts of SPs (1–2%). The achievement appears of great relevance if one considers that such volume fractions have negligible effect on ultimate tensile strength, and Young’s modulus [59] and conventional hardness tests are not considered reliable for revealing quantities of SPs below ≈ 4% [60,61,62]. For industrial applications, the importance of detecting such low amounts of SPs can be well understood by considering that the impact value of 2205 steel drops by about 50% in comparison with the original one (solution annealed and quenched steel) in the presence of ~1% of σ phase [63].

As expected, yield stress increases with the volume fraction of SPs; however, Figure 13 gives also other important information: the same amounts of SPs developed through heat treatments at 700, 850, and 900 °C correspond to different P_Y_/3 values. Specifically, the lower the temperature, the higher the yield stress. For example, a total SP amount of 10–11% gives rise to a yield stress of 672 MPa if the steel is treated at 750 °C, whereas in the case of treatment at 900 °C the value of P_Y_/3 is 622 MPa, i.e., 50 MPa lower. Such difference has to be ascribed mainly to the morphology of the σ phase, “coral-like” at 750 °C and “oil spot” at the higher temperatures considered in these experiments. This is consistent with the well-known behavior of DSSs which exhibit more brittle behavior after lower temperature precipitation [64] because in presence of the “coral-like” structure of the σ phase, even small strains lead to transcrystalline cracks with consequent toughness loss.

In conclusion, FIMEC allows one to reveal the precipitation of small SP amounts (~1%) not detectable through other mechanical tests and is also sensitive to the specific σ phase morphology (“coral-like” or “oil spot”); thus, it is a suitable technique for assessing in industrial practice the state of DSS after temperature exposure and the quality of welds.

## 4. Conclusions

The work reported some case studies where the FIMEC test has been successfully used to solve industrial problems regarding different metals and alloys. The results demonstrate its great versatility and capability to give information not available by employing conventional experimental techniques such as tensile, bending, and hardness tests.

The plastic behavior of a material under the penetration of a flat-ended indenter is today fully understood, and the theory gives an exhaustive description of its response in terms of stress vs. penetration depth, also taking into account the indentation size effect (ISE) phenomenon.

On these grounds, the FIMEC indentation test, which was originally developed for investigating the mechanical properties of irradiated materials, has proved to be a mature technique for a use on a large scale in industrial practice.

## Figures and Tables

**Table 1:** P_Y_/3 values obtained from FIMEC tests at different temperatures.

Temperature (°C)	25	100	200	300	400	500
P_Y_/3 (MPa)	794	615	575	488	425	410

**Table 2:** Nominal chemical composition of Eurofer-97 steel (wt%).

Cr	C	Si	Mn	P	S	Mo	W	V	Ta	Ti	N
8.87	0.10	0.05	0.45	0.005	0.004	0.0027	1.15	0.20	0.14	0.005	0.017
**Ni**	**Cu**	**Co**	**Al**	**Nb**	**B**	**O**	**As**	**Sn**	**Zr**	**Sb**	**Fe**
0.028	0.0035	0.006	0.008	0.0025	<0.001	0.0009	<0.005	<0.005	<0.005	<0.005	to balance

**Table 3:** Nominal chemical composition of the UNS 31803 steel and filling material (wt%).

	C	Cr	Cu	Mn	Mo	N	Ni	Si	P	S
UNS 31803	<0.03	21–23	­	<0.2	2.5–3.5	0.15–020	4.5–6.5	<0.1	<0.03	<0.02
Filling material	0.014	22.95	0.10	1.52	3.08	0.163	8.61	0.42	0.015	0.0008

**Table 4:** Micro-hardness HV and P_Y_/3 values determined by means of FIMEC tests in the positions shown in Figure 11. The HV/P_Y_/3 ratio is also shown.

Point	1	2	3	4	5	6	7	
Zone	HAZ	HAZ	MZ	HAZ	HAZ	MZ	HAZ	
P_Y_/3	607	637	628	637	607	662	620	
HV	278	275	284	274	289	290	285	
HV/ P_Y_/3	0.46	0.43	0.45	0.43	0.48	0.44	0.46	
**Point**	**8**	**9**	**10**	**11**	**12**	**13**	**14**	**A**
Zone	MZ	MZ	HAZ	MZ	MZ	MZ	MZ	BM
P_Y_/3	670	594	599	616	658	594	637	594
HV	290	289	256	275	280	279	283	256
HV/ P_Y_/3	0.43	0.48	0.43	0.44	0.43	0.47	0.44	0.43

**Table 5:** Nominal chemical composition of the 2205 steel (wt%).

C	Cr	Mn	Mo	N	Ni	Si	P	S	Fe
<0.03	21–23	<0.2	2.5–3.5	0.15–0.20	4.5–6.5	<0.1	<0.03	<0.02	to balance

## Data Availability

The data presented in this study are available on request from the corresponding author.

## Nomenclature

*R*	indenter tip radius
*r*	distance from the center of cylindrical indenter
*h*	indenter penetration depth
σ_z_	normal stress in z direction
τ*_rz_*	shear stress in the rz plane
*u* *_z_*	displacement in z direction
*P* *_m_*	mean contact pressure
*P* *_max_*	maximum contact pressure
*E*	Young’s modulus
*ν*	Poisson’s ratio
(r, θ, z)	local cylindrical coordinate system
*σ_r_*	normal stress in *r* direction
*σ_θ_*	normal stress in θ direction
*ξ*	shear strength
P_y_	upper limit of the first plastic stage of the experimental pressure-penetration curve
σ_y_	yield stress
*P*_0_	initial pressure in stress relaxation tests
*t*	time
τ	relaxation time in stress relaxation tests
FIMEC	Flat top Indenter for Mechanical Characterization
IFMIF	International Fusion Materials Irradiation Facility
CFRP	Carbon Fiber Reinforced Polymer
DBTT	ductile to brittle transition temperature
CRB	cracked Round Bar
ISE	indentation size effect
PS	plasma spray
*V* *_W_*	volume fraction of W in the graded layers between CuCrZr substrate and pure W coating
*x*	distance from the pure W coating
*t*	interlayer thickness
*p*	gradient index
TIG	tungsten inert gas
MIG	metal-arc inert gas
GTAW	gas tungsten arc welding
HAZ	heat affected zone
MZ	molten zone
PWHT	post welding heat treatment
TBM	Test Blanket Module of ITER
ITER	International Thermonuclear Experimental Reactor
DSS	duplex stainless steel
SP	second phase
SEM	scanning electron microscopy
TEM	transmission electron microscopy
